# Insights into monitoring changes in the viable cell density and cell physiology using scanning, multi-frequency dielectric spectroscopy

**DOI:** 10.1186/1753-6561-7-S6-P4

**Published:** 2013-12-04

**Authors:** John Carvell, Lisa Graham, Brandon Downey

**Affiliations:** 1 Aber Instruments Ltd, Abersytwyth, UK; 2 Bend Research Inc., Oregon, USA

## Background

Real-time bioprocess monitoring is fundamental for maximizing yield, improving efficiency and process reproducibility, minimizing costs, optimizing product quality, and full understanding of how a system works. The FDA's Process Analytical Technology initiative (PAT) encourages bioprocess workflows to operate under systems that provide timely, in-process results. At the same time the demand for ever increasing supplies of biological pharmaceuticals, such as antibodies and recombinant proteins, has fueled interest in streamlined manufacturing solutions. Bioreactors that are monitored continuously and in real-time offer the advantage of meeting current and future supply demands with biological product of the utmost quality and safety, achieved at the lowest overall cost and with least risk. This paper will focus on how one research groups in has used scanning multi-frequency dielectric spectroscopy to comparatively profile multiple bioreactor runs and elucidate fine details concerning cell viability and mechanism of cell death. The cellular information observed has not been available through other technologies. The presentation will also focus on how the technology can also be applied to Single use Bioreactors in a cGMP environment and on samples down to 1 ml volume.

## Introduction

• Dielectric spectroscopy (DS) is now the most common method for estimating the *in situ *live cell concentration in animal cell culture.

• DS and traditional offline methods for cell counting based on Trypan Blue correlate well during the growth phases but with some cell lines, deviations are observed during the late growth phase.

• Scanning multi-frequency DS can detect the physiological changes of the cells during the death phase of the culture including changes in cell size, membrane capacitance and internal conductivity [1-3].

• The concept of using the Area Ratio Algorithm (ARA) looks to be a relatively simple and promising method for providing on-line cell counts that correlate well with traditional methods for the complete cell growth cycle.

## Background of DS and the Futura Biomass Monitor

• DS measures the passive electrical properties of cells in suspension through the cells' interaction with RF excitations.

• Viable cells are composed of a conducting cytoplasm surrounded by a non-conducting membrane suspended in a conducting medium. When an alternating current is applied to the suspension, each cell becomes polarised and behaves electrically as a tiny spherical capacitor.

• The suspensions reaction to the current is expressed as its permittivity can be measured by its capacitance and conductivity as a function of frequency. Viable cells possess intact membranes which prevent the free flow of ions and allow the cells to polarise. Dead, porous cells and debris lack an enclosing membrane and are unable to build up charge separation. Hence, DS measures only viable cells.

• The Futura Biomass Monitor (Aber Instruments Ltd, UK) measures the capacitance created directly from the cells. The capacitance signature of cells is measured between 50 KHz and 20 MHz with readings every 30 seconds.

• At low excitation frequencies the cells can fully polarise and the capacitance of the suspension is maximised. As the excitation increases, the cells lose their ability to fully polarize and the measured capacitance drops eventually measuring no polarisation at high frequencies.

## Concept of the Area Ratio algorithm

• A novel method for obtaining an enhanced prediction of viable cell volume fraction (VCV) compared to currently employed methods has been developed, wherein changes in cell health are quantified using frequency scanning data. In the novel method, cell health is measured by using an area ratio (AR) to quantify the shape of the measured dielectric spectrum using the following algorithm:

(1) $$\begin{array}{c} AR=\frac{\overset{f_{H}}{\underset{f_{Q}}{\int }}C\left(f\right)df}{\overset{f_{H}}{\underset{f_{L}}{\int }}C\left(f\right)df}\\ f_{H}<f_{Q}<f_{L} \end{array}$$

Where:

**AR **= area ratio for a given scan

**f_H _**= highest frequency of the scan

**f_L _**= lowest frequency of the scan

**f_Q _**= semi arbitrary chosen frequency between f_H _and f_L_

**C(f) **= capacitance as a function of frequency

• The AR is used as a correction factor to correct for the death phase divergence in the following manner:

(2) $$VCV\left(t\right)=A\times \left(C\left(t\right)-B\times AR\left(t\right)-k_{2}\right)+k_{1}$$

Where:

**VCV **= predicted viable cell volume fraction

**A and B **= fit constants of proportionality relating dielectric measurements to offline cell measurements

**k_1 _and k_2 _**= constant offset values

**Figure 1:**
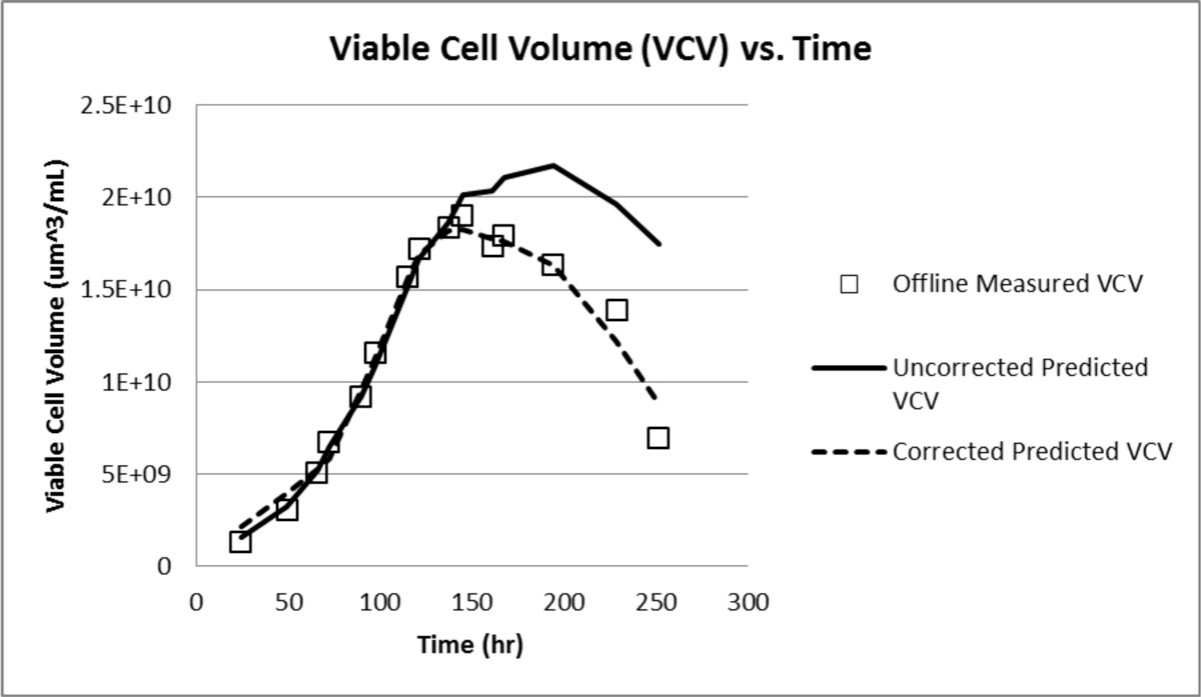
**Implementation of the Area Ratio Algorithm (ARA) yields enhanced prediction of viable cell volume fraction compared with uncorrected methods**.

• Changes in cell health are quantified using frequency scanning data. When the ARA is applied to the uncorrected VCV derived from the capacitance data, there is a good match with the off-line derived VCV (Figure 1).

## Applying multi-frequency scanning DS to single use bioreactors and samples off-line

• A single use sensor has been developed by Aber Instruments and the early versions utilized stainless steel electrodes. This sensor was suitable for single or dual frequency DS and the performance has been compared with traditional probes that are used on reusable bioreactors [4].

• Samples as low as 100 microlitre can be withdrawn from a bioreactor and scanning DS can be applied using existing DS probes. An example of this is shown in the full version of the poster with distinctly different frequency scans for healthy and unhealthy cells. The unhealthy cells were generated by treatment with 1 uM staurosporine to induce apoptosis.

## Discussions and conclusions

The work presented here shows the utility of frequency scanning data to obtain enhanced measurement of VCV using non-invasive capacitance sensors in reusable and single use bioreactors. The information-rich nature of dielectric frequency scanning allows interrogation of biophysical properties of cells. The concept can be extended to samples off-line.
